# Pediatric Cardiologist's View on Centralization of Pediatric Cardiology Services: A Questionnaire Survey

**DOI:** 10.3389/fcvm.2021.769923

**Published:** 2022-02-11

**Authors:** Omar R. Tamimi, Omer A. AlGonaid, Yahya H. AlMashham, Abdulrahman Y. Almashham

**Affiliations:** King Fahad Medical City, Riyadh, Saudi Arabia

**Keywords:** pediatric cardiology services, centralization, regionalization, birth rate, waiting list, patient load, quality, Saudi Arabia

## Abstract

The population of the Kingdom of Saudi Arabia (KSA) exceeding 35 million people and in the presence of a non-structured increase in the number of pediatric cardiac centers, we expect to face some concerns like dilution of the service where the volume will be less than the acceptable standards, the increase in mortality and morbidity, and failure to obtain personalized medicine at a reasonable cost. Therefore, we built up this survey questionnaire about those concerns and collected the opinion of expert medical staff in Saudi Arabia who are working in the field of pediatric cardiology. Seventy percent of the responders vs. 25% recommend the centralization of the PCS as the solution for the above concerns, and 94% recommend sticking to the globally accepted criteria when issuing the license of the centers providing PCS including the volume of patients in each center, and minimum multidisciplinary facilities in terms of resources, services, and personnel.

## Introduction

The population of the Kingdom of Saudi Arabia (KSA) exceeds 35 million people (1); however, the birth rate continues to show a decreasing pattern in the recent report of the World Bank (2).

The recently reported prevalence of congenital heart disease in KSA looks to be higher than reported in other populations in the Middle East and Europe (3). We explain that this relative prevalence increase, when compared to neighboring countries, is due to an increase in PCS in a well-structured system, while when compared with European countries it might be related to higher inbreeding and fertility in KSA compared to European countries. In the presence of a non-structured increase in these centers, we expect to face the same global issues that emerged in comparable situations (4), those issues including the concern of dilution of the service where the volume will be less than the acceptable standards (5), the increase in mortality and morbidity (6), and failure to maintain quality medicine at a reasonable cost (7). The institution of more centers mandates the presence of all comprehensive hospital practices that must run 24/7/365. These include all levels of urgency as well as all other specialties of the hospital system (including radiology, laboratory medicine, pharmacy, and blood bank) (4), which could represent an unreasonable load to the health system. These concerns are studied in many developed countries and they consider the centralization of the PCS to a few centers a practical solution for these concerns (6–8).

The concept “centralization” in our survey refers to a process of concentration of resources, which includes sophisticated infrastructures, qualified staff, well-equipped materials, continuously updated knowledge, and a well-structured research system. This process includes the globalization of low volume centers into fewer but highly specialized and larger volume centers distributed in 5 provinces in KSA within the so-called concept of “regionalization” (4). These 5 major centers offer all non-interventional and almost all interventional PCS for their own citizens except for very complicated surgeries and interventions such as Norwood surgery, ECMO, and heart transplantation are kept in the capital city (Riyadh) or in addition to Jeddah.

The process of centralization should keep the current basic pediatric cardiology services (clinical/echocardiography) in the peripheral cities of KSA and keep an open referral channel for advanced PCS in these proposed 5 major centers.

Therefore, we build up this survey questionnaire about those concerns, to collect the opinion of expert medical staff in Saudi Arabia who are working in the field of pediatric cardiology.

## Method

Using a secured Internet-based survey (google forms at www.google.com, see attachment 1) we used this form to collect the responses as well as to extract the results and data analysis.

From the total of 175 licensed consultants (cardiologists and surgeons), we sent the Google form to 150 consultants, and 106 responded (95 pediatric cardiology consultants and 11 pediatric cardiac surgeon consultants).

## Results

Data are presented as percentages for nominal variables, or ranges for continuous variables.

### Demographics

The questionnaire was sent to 150 PCC/PCSC, of whom 106 responded to the questionnaire, 95 were pediatric cardiologists, and 11 were pediatric cardiac surgeons. The response rate was 71% among the PCC/PCSC.

The summary of demographics is illustrated in Table 1 and the summary of the answers is illustrated in Table 2.

**Table 1:** Showed the participants characters regarding their field of practice and also the length of experience.

Specialty	90.5% (95 participants)	9.5% (11 participants)
	Pediatric Cardiologists	Pediatric Cardiology surgeons
Experience	33% (35 participants)	67% (71 participants)
	<10 years	>10 years

*It showing clearly most of them has long experience in the field*.

**Table 2:** Showing the summary of the participant responses to our main survey questions.

**Question**	**Yes**	**No**	**Don't know**
Do you think we should do centralization?	70% (75)	22% (23)	8% (8)
Do we need more centers?	25% (26)	70% (73)	5% (5)
With changes in health sector due to health transformation plan, are there centers which will close?	46% (49)	24% (25)	30% (32)
Is it ok for any hospital to provide the service regardless of the number of criteria?	4% (4)	94% (100)	2% (2)
Is the waiting list coming down?	71% (75)	24% (26)	5% (5)
Is the number of new cases coming down?	56% (59)	43% (46)	2% (2)
Is the birth rate in Saudi Arabia coming down?	41% (43)	39% (42)	20% (21)
Are the fellows exposed to enough numbers?	41% (43)	38% (40)	21% (22)

Most of the responders (67%) had completed more than 10 years of experience in the field of pediatric cardiology.

### The Pediatric Cardiology Service Centralization

Among the responders, the majority (70%) had considered doing centralization of the PCS in the centers of the main 5 KSA provinces as a strategic plan and the same percentage declared no need for the institution of further centers. Moreover, about 46% thought some centers would be closed due to the health transformation plan. The vast majority (94%) strictly recommended sticking to the globally accepted criteria when issuing the license of the centers providing PCS. About 71% of the responders thought that the patient's waiting lists were coming down, and 56% of the responders mentioned that the lists of new cases were also coming down. A total of 41% were aware that the birth rate in KSA is decreasing, whereas 39% thought that it is increasing.

The summary of these responses is illustrated in Table 2.

## Discussion

The majority (70%) of responders expect that the centralization of PCS in the centers of each province will improve the outcome of these services and decrease morbidity and mortality as it was proved in many registries and reports in Europe (6) and the United States (4). For example, in Sweden, regionalization of care from 4 to 2 centers was associated with a decline in surgical mortality from 9.5 to 1.9% (6). The participants clearly stated that there was no need for more PCS centers and 46% of the responders were thinking in advance and expecting that some centers might be globalized especially with the health transformation strategy adopted by the ministry of health in KSA, which is going in harmony with the discussed survey in the centralization of PCS.

As the vast majority of the responders (94%) strongly recommend applying the globally accepted criteria (9) for any centers providing PCS, therefore the only way to achieve this recommendation is to centralize these services to a few centers that are distributed according to population density. In 2011, the United Kingdom-Bristol Royal Infirmary inquiry included a massive survey and review study of physicians, hospitals, the public, and policymakers. It resulted in the recommendation to decrease the number of centers providing PCS from 11 to 7 in order to improve the quality of care (10). In 2016, the review of the changes found that health care outcomes were improved (9). Despite an annual increase in the number of cases operated between 2000 and 2009 from 2,283 to 3,939, the 30-day death rate dropped from 4.3 to 2.6% of cases (9). The improvement extended to cover several areas of care, for example, numbers of medical surgical and nursing staff, patient satisfaction, facilities expansion, complex surgical procedure addition, and others (9).

On the other hand, the trainees should be exposed to a good volume of patients and procedures to produce a good outcome in pediatric cardiology training programs (5). The latter principle is a rapidly growing important criterion for any center providing PCS. The Bristol Royal Infirmary inquiry report documented inadequate standards of care and a higher mortality rate at one center (10) due to deficiency of some accreditation criteria including the appropriate volume of patients. However, in our survey, only 41% of the responders stated that the trainees are exposed to acceptable patient volume. Therefore, this will also be another supportive evidence that makes centralization of PCS as a gold standard to improve the outcome in training programs.

The centralization of PCS could serve as a model for the coordination of other low volumes, such as high complexity specialties (11).

## Strength and Limitations

One of the novel aspects of this paper is to bring light into understanding the current situation of the pediatric cardiac service in KSA through the opinions of experienced medical staff in the field of PCS. Such opinions could be used in better planning for the future of the pediatric cardiac service in KSA.

As this survey depends on personal experience, which represents subjective rather than objective judgments, this could be considered as a point of weakness. However, the results of this survey are supported by well-standardized international studies in different countries (4, 6–8), as well as could be considered a pilot introduction to produce more national and regional objective evidence in the future.

## Conclusion

Centralization of PCS to reasonably limited centers within the main provinces according to population density will provide specific health assistance in the pediatric cardiology field in KSA and other Middle Eastern countries. This method might produce a good outcome for pediatric cardiology, training programs, and research background as well as a cost-effective strategy and a quality improvement tool to provide PCS. This conclusion is supported by the results of our survey questionnaire and by well-standardized international studies as well.

## Data Availability Statement

The raw data supporting the conclusions of this article will be made available by the authors, without undue reservation.

## Ethics Statement

The studies involving human participants were reviewed and approved by King Fahad Medical City, Institutional review board with IRB and FWA registration numbers: IRB00010471 and FWA00018774. The patients/participants provided their written informed consent to participate in this study.

## Author Contributions

All authors listed have made a substantial, direct, and intellectual contribution to the work and approved it for publication.

## Conflict of Interest

The authors declare that the research was conducted in the absence of any commercial or financial relationships that could be construed as a potential conflict of interest.

## Publisher's Note

All claims expressed in this article are solely those of the authors and do not necessarily represent those of their affiliated organizations, or those of the publisher, the editors and the reviewers. Any product that may be evaluated in this article, or claim that may be made by its manufacturer, is not guaranteed or endorsed by the publisher.

## Abbreviations

KSA: Kingdom of Saudi Arabia
PCC/PCSC: pediatric cardiology consultants and pediatric cardiac surgeons consultants
PCS: pediatric cardiology services.
